# Evaluating the Utility of Single-Locus DNA Barcoding for the Identification of Ribbon Worms (Phylum Nemertea)

**DOI:** 10.1371/journal.pone.0155541

**Published:** 2016-05-12

**Authors:** Per Sundberg, Sebastian Kvist, Malin Strand

**Affiliations:** 1 Department of Marine Science, University of Gothenburg, Box 463, SE-405 30, Gothenburg, Sweden; 2 Department of Natural History, Royal Ontario Museum, 100 Queen’s Park, Toronto, ON, M5S 2C6, Canada; 3 Department of Ecology and Evolutionary Biology, University of Toronto, 25 Willcocks Street, Toronto, ON, M5S 2B4, Canada; 4 Swedish Species Information Center, Swedish University of Agricultural Sciences, SE-75007, Uppsala, Sweden; Consiglio Nazionale delle Ricerche (CNR), ITALY

## Abstract

Whereas many nemerteans (ribbon worms; phylum Nemertea) can be identified from external characters if observed alive, many are still problematic. When it comes to preserved specimens (as in e.g. marine inventories), there is a particular need for specimen identifier alternatives. Here, we evaluate the utility of COI (cytochrome *c* oxidase subunit I) as a single-locus barcoding gene. We sequenced, data mined, and compared gene fragments of COI for 915 individuals representing 161 unique taxonomic labels for 71 genera, and subjected different constellations of these to both distance-based and character-based DNA barcoding approaches, as well as species delimitation analyses. We searched for the presence or absence of a barcoding gap at different taxonomic levels (phylum, subclass, family and genus) in an attempt to understand at what level a putative barcoding gap presents itself. This was performed both using the taxonomic labels as species predictors and using objectively inferred species boundaries recovered from our species delimitation analyses. Our data suggest that COI works as a species identifier for most groups within the phylum, but also that COI data are obscured by misidentifications in sequence databases. Further, our results suggest that the number of predicted species within the dataset is (in some cases substantially) higher than the number of unique taxonomic labels—this highlights the presence of several cryptic lineages within well-established taxa and underscores the urgency of an updated taxonomic backbone for the phylum.

## Introduction

Taxon identification is a fundamental part of taxonomy, systematics, ecology, and biodiversity research. Identification of metazoans is traditionally based on morphological diagnoses, which requires special identification tools and competence acquired through extensive experience and training. For several taxonomic groups, this may also entail specialized techniques and equipment, such as histological sectioning and microscopy. This is true for the phylum Nemertea, a group of soft-bodied, unsegmented worm-like animals that range in length from a few millimeters up to 30 meters [1,2]–their body width rarely exceeds a few millimeters. Most known species of nemerteans are marine, although some species have adapted to freshwater or terrestrial (often semi-aquatic) habitats (e.g. [3,4]). Nemerteans do not possess any external body appendages, and the external diagnostic characters are often restricted to size, shape, position of mouth and proboscis pore, number and pattern of eyes, and coloration. It has often been repeated in the literature that the identification of most nemertean species is demanding and requires the “study of internal anatomy by means of light microscopy and serial sections”[5]. Contrary to this, our experience is that many species in fact can be identified from combinations of external characters (color, size, eye number and pattern, cephalic furrows, body shape). But, this requires that the animals be studied alive, which is impossible in many cases, particularly concerning marine inventories where identification is often based on bulk fixed specimens. In addition, very few investigators are likely to go through the process of sectioning animals to find characters for the purpose of identification. More likely, the specimens will be reported simply as “Nemertea sp.” (see e.g. [6]). Furthermore, there is no scientific evidence that species identifications are more accurate when based on internal characters, as opposed to external, and Strand et al. [7] also pointed out the fallacy of this approach.

To this end, we emphasize the importance of distinguishing between description and identification—we are not opposing species delimitation/descriptions based on internal anatomy and systematic conclusions drawn from this information. However, one should be aware of the fact that several morphological characters historically used to describe species show high levels of plasticity within single species (see [8]). That is, several characters thought to be important in diagnosing both species and genera of nemerteans show high levels of intraspecific variation [9]. Conversely, when it comes to the species-level identification of a sampled specimen based solely on internal anatomy, it has already been shown that the characters commonly used often overlap even between species from (putatively) different genera [7].

We conclude that there is a need for an alternative to traditional, morphology-based approaches when it comes to accurate and rapid identifications of nemerteans. The problem of species identifications is of course not only relevant for nemerteans, but also for other taxa. See for example Haase et al. [10] regarding the effect of misidentifications when it comes to precision in monitoring programs. The most promising approach for this is to employ molecular data that complement or replace morphological data, and the consortium for the Barcode of Life (CBoL) has agreed on using a 658 base-pair fragment at the 5´-end of the mitochondrial cytochrome *c* oxidase subunit I gene region (COI) as a default barcode region for metazoans [11]. While this gene region has been successfully used as a DNA barcode for a variety of metazoan groups, the interspecific COI divergence is too low to fulfill the objective of providing reliable identifications in some taxa [12,13], which may lead to type I or type II errors [14–16]. The necessary gap between the maximum intraspecific and minimum interspecific divergence is typically referred to as a barcoding gap and considered imperative for accurate and effective barcoding (e.g. [17–19]). It has been suggested, however, that such distance threshold boundaries are not suitable for specimen identifications [20,21], mainly because rates of evolution within metazoan mitochondrial genomes have been shown to vary substantially between interspecific and intraspecific comparisons, as well as between different groups of species [20,22]. As a result, an alternative means of barcoding, relying instead on diagnostic character states, has emerged and has already been successfully applied to numerous animal groups [23–26].

In the present study, we aim to test if COI is useful as a standard barcode for nemerteans. For some problematic nemertean clades, we contrast a distance-based with a character-based approach (CAOS) to illuminate the opportunity for accurate character-based DNA barcoding, even in the absence of a distinct and sufficiently wide barcoding gap. We also contrast results from several species delimitation analyses with calculated intraspecific and interspecific genetic variation values in order to assess whether or not a standard cutoff for within-species variation can be formalized for Nemertea, and investigate if a barcoding gap presents itself when species affiliations are more objectively assigned.

## Materials and Methods

### Sequence data

Our analyses are based on both GenBank sequence data and new data from over 500 nemerteans collected over several years and from numerous localities, spanning continents (most often collected by PS and MS). Field permits for collecting marine invertebrate specimens was granted to Per Sundberg from various governing bodies. The sampling, collecting sites, and DNA extraction/sequencing procedures are described elsewhere, in various contributions (e.g. [7,27]). Briefly, COI sequences were downloaded for all nemertean taxa present on GenBank (http://www.ncbi.nlm.nih.gov/genbank/) and the newly generated sequences were added to this pool. The DNA dataset was restricted to sites for which comparative data was available for all included specimens—sites with multiple leading and lagging gaps were deleted from the dataset.

### Distance-based barcoding and the barcoding gap

The methods used for detection of barcoding gaps follow Kvist [28]. To enable robust *in silico* separation of comparisons into intraspecific and interspecific bins, sequences lacking species-level identifications were purged from the dataset. Imprecise taxonomic labels (e.g. “*Micrura* sp.”) were excluded because comparisons using these sequences cannot with certainty be funneled into either of these bins. Eleven different datasets were compiled from the full dataset by selecting sequences for four different taxonomic levels (phylum, subclass, family and genus), imposing the criteria that each of these needed to include at least 100 sequences for more than three different nominal species. At the genus level, only one taxon fulfilled these criteria (*Oerstedia*) and, for comparative purposes, we therefore also included additional datasets at this taxonomic level (*Cerebratulus*, *Lineus* and *Micrura*) that did not meet these criteria. The three latter genera were chosen because they have previously been referred to as “mega-genera” [29,30] and will likely represent a worst-case scenario regarding the presence of a sufficiently sized barcoding gap. The division of the full dataset was performed bearing in mind that enough comparative data was needed to make solid inferences with regard to the presence or absence of a barcoding gap, and to enable comparative analyses between taxonomic levels in order to assess the presence or absence of global, as well as local barcoding gaps. In other words, the focus here was to increase our understanding regarding at which taxonomic level a putative barcoding gap presents itself. Consequently, the 11 datasets included all sequences of representatives of the following taxa: (1) Nemertea, (2) Heteronemertea, (3) Hoplonemertea, (4) Palaeonemertea, (5) Lineidae, (6) Cephalotrichidae, (7) Oerstediidae, (8) *Oerstedia*, (9) *Cerebratulus*, (10) *Lineus* and (11) *Micrura*. Datasets ranged from 32 sequences to 915 sequences. Each dataset was separately aligned using MAFFT ver. 7 [31] employing the G-INS-i strategy, which is suggested for >200 sequenced with global homology motifs (note that no gaps were present in the resulting alignment). Mesquite v. 2.5 [32] was then used to create nexus files from the alignments and COI distances were calculated in PAUP* v. 4.0d98 [33]. Following the results of Srivathsan & Meier [34], uncorrected *p* intra- and interspecific distances were calculated under the function of minimal evolution, ignoring gaps for affected sites, constraining branch lengths to be non-negative, with equal rates for variable sites, and estimating variation for all substitutions. Distances were thereafter divided into intraspecific and interspecific bins using the commands detailed in Kvist [28] and Microsoft Excel was used to create graphs from the comparisons.

### Character-based barcoding

As a complement to the distance-based approach, character-based barcoding was applied to genus-level groups via the Characteristic Attributes Organization System (CAOS) [23] (http://bol.uvm.edu/caos-workbench/caos.php). This was to investigate whether or not the nominal species that showed high intraspecific distances (2% and above) still possessed diagnostic character states within the COI sequences that may allow for accurate identifications despite the lack of a clear barcoding gap for these sets of sequences. Importantly, representatives for each lineage were compared to sequences both from within the same nominal species (local comparison) and also to the full set of COI sequences (global comparison). Initially, distinct lineages within these putative species complexes were identified through a neighbor joining analysis conducted in PAUP* with the same settings as for the distance-based analyses and diagnostic characters (or characteristic attributes;CA’s) were assessed for each lineage that contained more than six specimens—this to provide statistical power to the analysis.

### Estimating the number of OTUs

We used three complementary strategies to assess the number of OTUs (Operational Taxonomic Units; i.e., putative species) within the dataset. First, using an ultrametric tree recovered from BEAST ver. 1.8.3 [35] under the HKY model of nucleotide evolution, estimated base frequencies, a gamma model for site heterogeneity, and an uncorrelated relaxed clock, we applied a General Mixed Yule Coalescent model (GMYC) [36] using the GMYC web server on the Exelixis lab website (http://species.h-its.org/gmyc/). For this purpose, both single and multiple threshold models were used. Second, we used a phylogenetic tree recovered from a standard search strategy (GTR + G model of sequence evolution, 1000 iterations with 25 initial GAMMA rate categories and final optimization with four GAMMA shape categories) in RAxML [37] and this was used as the input for a Poisson Tree Processes analysis (PTP) [38] on the online PTP server at http://species.h-its.org/ptp/. The PTP analysis employed 500,000 MCMC generations (the maximum allowed) with a thinning value of 100 and 25% burn-in. Finally, we used statistical parsimony network analyses in TCS ver. 1.21 [39] to estimate the number of OTUs within the dataset. In agreement with Hart & Sunday’s [40] assumptions, we set the connection limits to 95% and 98%. The rationale behind these approaches was to compare the resulting number of OTUs with the number of taxonomic labels present in the dataset and to increase our knowledge regarding a general level of genetic variation at which intraspecific comparisons and interspecific comparisons are separated.

## Results

The final dataset is available from TreeBase (https://treebase.org/treebase-web/home.html) under submission ID 18972, and the newly generated COI sequences are deposited at GenBank under accession numbers KU839732-KU840166, KU840171-KU840188, KU840190-KU840206, KU840208-KU840223 and KU840225-KU840290 (see S1 Table). The final dataset consisted of 513 aligned nucleotide positions (no gaps were present) for 915 individual COI sequences representing 161 unique taxonomic labels (i.e., putative species) for 71 genera.

### Barcoding gap

#### Nemertea

**Fig 1:**
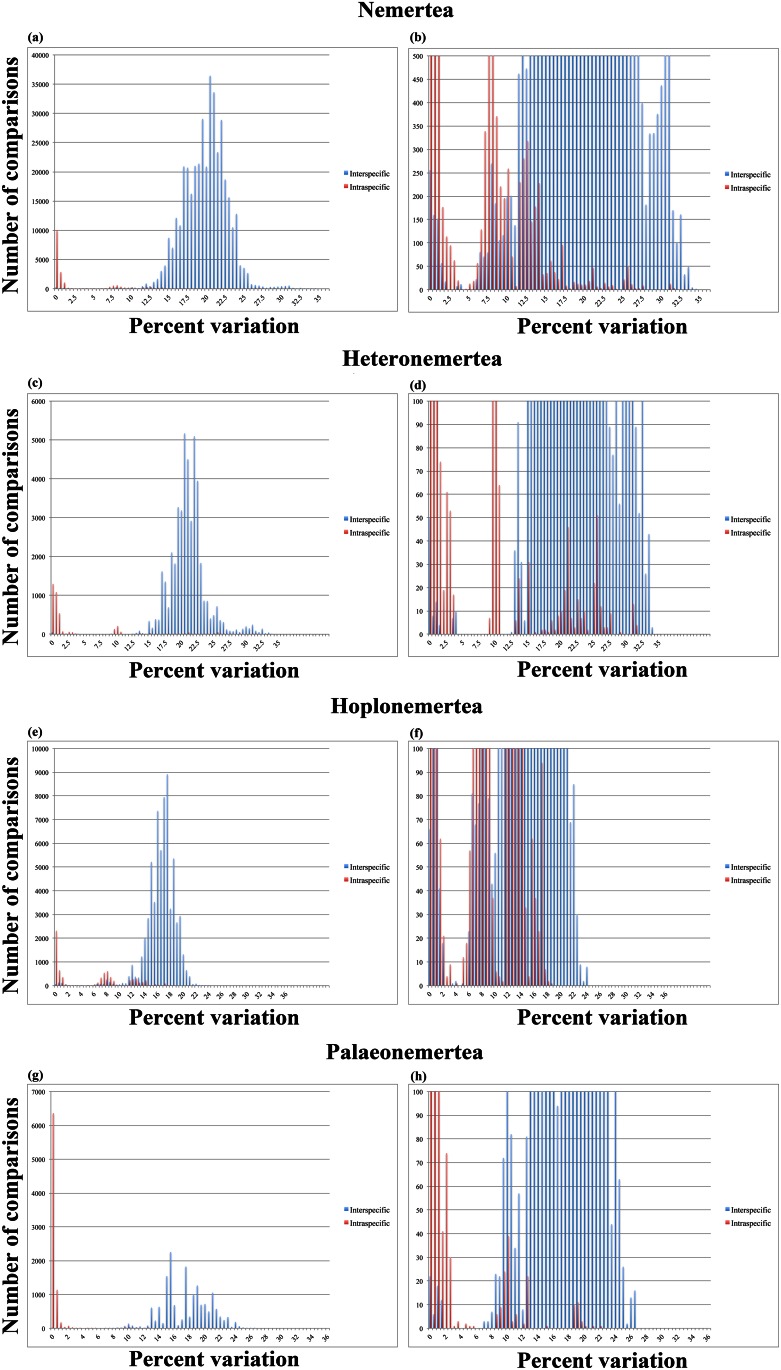
Result from the manual calculations of intraspecific (red) and interspecific (blue) COI distances. Note the absences of a disjunction between intraspecific and interspecific distances (the lack of a barcoding gap), which is further discussed in the text. A, Nemertea, full view of the chart with the x-axis set above the upper limit of the number of comparisons within the dataset; B, Nemertea, enlarged view of the barcoding gap region with the x-axis set to a maximum of 500 comparisons; C, Heteronemertea, full view of the chart with the x-axis set above the upper limit of the number of comparisons within the dataset; D, Heteronemertea, enlarged view of the barcoding gap region with the x-axis set to a maximum of 100 comparisons; E, Hoplonemertea, full view of the chart with the x-axis set above the upper limit of the number of comparisons within the dataset; F, Hoplonemertea, enlarged view of the barcoding gap region with the x-axis set to a maximum of 100 comparisons; G, Palaeonemertea, full view of the chart with the x-axis set above the upper limit of the number of comparisons within the dataset; H, Palaeonemertea, enlarged view of the barcoding gap region with the x-axis set to a maximum of 100 comparisons.

The distance-based analysis for the entire pool of nemertean sequences resulted in 398,957 interspecific comparisons and 19,200 intraspecific comparisons. Whereas the resulting graph showing the full range of comparisons on the Y-axis (Fig 1a) seems to indicate the presence of a distinct barcoding gap, a more focused view of the gap itself (Fig 1b) shows a dip in the number of comparisons at about 4%-5% variation, which is bordered on both sides by both interspecific and intraspecific comparisons. As such, only a narrow barcoding gap seems to exist between sequences of the full dataset. In total, 644 interspecific comparisons (0.2%) show between 0% and 2% genetic distances, whereas 4,784 intraspecific comparisons (24.9%) show genetic distances above 5%.

#### Heteronemertea

Out of the 48,518 comparisons that were conducted for heteronemertean taxa, 44,633 were interspecific comparisons and 3,885 were intraspecific comparisons. The results (Fig 1c and 1d) suggest that there is a barcoding gap present between 4–9% variation within the dataset (the number of comparisons within this range reaches 0). However, much like the results from the full dataset (see above), this gap-region is flanked on both sides by both interspecific and intraspecific distances, resulting in the functional inadequacy of the “gap” present between 4–9%. Notably, a conspicuous peak in intraspecific comparisons is present between 9–10.5%, which solely involves intraspecific comparisons of *Parborlasia corrugatus* (McIntosh, 1876). For the isolated Heteronemertea dataset, only 77 interspecific comparisons (0.2%) fall within the range of 0–2% distance, while 738 intraspecific comparisons (19.0%) show distances above 5%.

#### Hoplonemertea

The dataset including only representatives of Hoplonemertea resulted in 65,046 interspecific comparisons and 7,346 intraspecific comparisons. The distance-based results follow the trend in the previous datasets inasmuch as the absence of a barcoding gap (Fig 1e and 1f) is the result of the placement of both interspecific and intraspecific comparisons on either side of the, albeit relatively narrowly distributed, “barcoding gap” (between about 4–5%). Interestingly, there are two main peaks of intraspecific variation that lie above 6% variation. Both of these peaks (at 6–9.5% and 11.5–14.5%, respectively) involve intraspecific comparisons between *Oerstedia dorsalis* (Abildgaard, 1806), as well as *Oerstedia striata* Sundberg, 1988, with the exceptions of a few within-species comparison for *Emplectonema buergeri* Coe, 1901, *Tetrastemma peltatum* Bürger, 1895, *T*. *candidum* (Müller, 1774), *T*. *robertianae* McIntosh, 1874 and *T*. *flavidum* Ehrenberg, 1828. In total, 383 interspecific comparisons (0.6%) show distance values of 2% or below, whereas fully 3,917 (53.3%) intraspecific comparisons show distance values of above 5% (almost all of these involve *O*. *dorsalis* and *O*. *striata*).

#### Palaeonemertea

16,341 interspecific comparisons and 7,971 intraspecific comparisons were performed for palaeonemertean taxa. Judging from the resulting graphs (Fig 1g and 1h), this dataset seems to behave in a more unproblematic sense regarding the barcoding gap. This is mainly due to two factors: first, there are relatively few intraspecific comparisons that fall above 5% and, second, a proportionally smaller amount of comparisons fall within the range of where a barcoding gap is expected to present itself (2–8%). However, the “gap” is again surrounded on both sides by interspecific and intraspecific distances such that no clear separation of the different types of comparisons is present. With the exception of a single comparison for *Carinoma tremaphoros* Thompson, 1900, the intraspecific comparisons that show distance values of above 5% (n = 140; 1.8%) involve species of *Cephalothrix*, in particular *C*. *simula* Iwata, 1952 and *C*. *spiralis* Coe, 1930. Likewise, the few interspecific comparisons that lie below 2% (n = 59; 0.4%) also involve species of *Cephalothrix*. As it is highly unlikely that the rate of COI evolution is relatively increased in narrow parts of the genus and decreased in other parts, this result seems to instead suggest that this taxon is particularly difficult to accurately ID.

#### Lineidae

**Fig 2:**
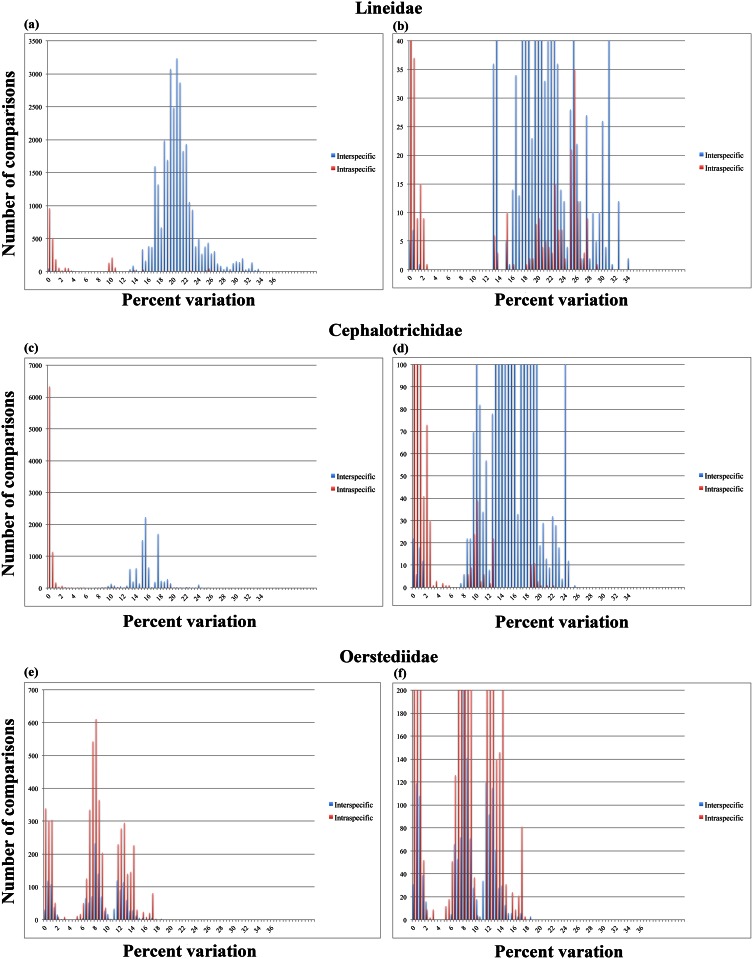
Result from the manual calculations of intraspecific (red) and interspecific (blue) COI distances. A, Lineidae, full view of the chart with the x-axis set above the upper limit of the number of comparisons within the dataset; B, Lineidae, enlarged view of the barcoding gap region with the x-axis set to a maximum of 40 comparisons; C, Cephalotrichidae, full view of the chart with the x-axis set above the upper limit of the number of comparisons within the dataset; D, Cephalotrichidae, enlarged view of the barcoding gap region with the x-axis set to a maximum of 100 comparisons; E, Oerstediidae, full view of the chart with the x-axis set above the upper limit of the number of comparisons within the dataset; F, Oerstediidae, enlarged view of the barcoding gap region with the x-axis set to a maximum of 200 comparisons.

For representatives of the family Lineidae, the distance-based analyses resulted in a pool comprised of 30,083 interspecific and 2,559 intraspecific comparisons. Unlike the previous taxa, the resulting graphs for Lineidae shows a clearer separation between intraspecific and interspecific comparisons, such as the one expected when a barcoding gap is present (Fig 2a and 2b). However, a strict barcoding gap still does not exist as both types of comparisons again border the discontinuation. In this case, 77 interspecific comparisons (0.3%) display distance values of below 2%, whereas 688 intraspecific comparisons (26.9%) show distances above 5%.

#### Cephalotrichidae

The results for Cephalotrichidae consisted of 9,641 interspecific comparisons and 7,939 intraspecific comparisons. The graph strongly resembles that of Lineidae in that intraspecific and interspecific distances are relatively clearly, but not fully, separated from each other by a discontinuation in the range of genetic distances (Fig 2c and 2d). A total of 59 interspecific comparisons (0.6%) resulted in distances values below 2% and 139 intraspecific comparisons (1.8%) showed values above 5%.

#### Oerstediidae

Oerstediidae proved to be the most problematic taxon with respect to the absence of a barcoding gap as the interspecific comparisons (n = 1,521) completely overlap with the intraspecific comparisons (n = 4,809) in terms of distance values (Fig 2e and 2f). Notwithstanding that this dataset included sequences for six unique taxonomic labels, it was completely dominated by *Oerstedia dorsalis*, which may be the reason for the complete lack of a barcoding gap. Nevertheless, fully 314 interspecific comparisons (20.6%) showed distance values below 2% and 3,790 intraspecific comparisons (78.8%) resulted in distances above 5%.

**Oerstedia:** The dataset for *Oerstedia* was identical to that of Oerstediidae and, thus, the results conveyed above for Oerstediidae are identical to those for this dataset (results not shown).

**Fig 3:**
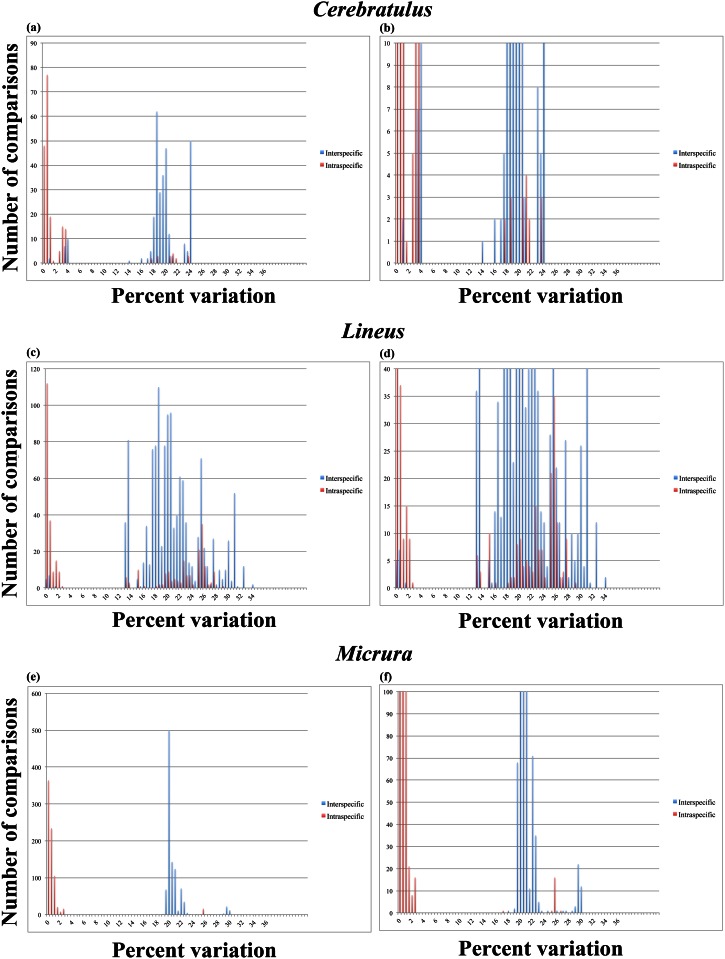
Result from the manual calculations of intraspecific (red) and interspecific (blue) COI distances. Note the absences of a disjunction between intraspecific and interspecific distances (the lack of a barcoding gap), which is further discussed in the text. A, *Cerebratulus*, full view of the chart with the x-axis set above the upper limit of the number of comparisons within the dataset; B, *Cerebratulus*, enlarged view of the barcoding gap region with the x-axis set to a maximum of 10 comparisons; C, *Lineus*, full view of the chart with the x-axis set above the upper limit of the number of comparisons within the dataset; D, *Lineus*, enlarged view of the barcoding gap region with the x-axis set to a maximum of 40 comparisons; E, *Micrura*, full view of the chart with the x-axis set above the upper limit of the number of comparisons within the dataset; F, *Micrura*, enlarged view of the barcoding gap region with the x-axis set to a maximum of 100 comparisons.

**Cerebratulus:** The dataset for sequences of *Cerebratulus* was represented by 301 interspecific and 197 intraspecific comparisons. The resulting graph (Fig 3a and 3b) shows a distinct gap between ~4–14%, indicating that a barcoding gap could indeed be present for this taxon. However, there is again flanking on both sides of the gap by both interspecific and intraspecific comparisons. All of the intraspecific comparisons that showed percentages of variation greater than 3.5% uncorrected *p* distance (starting at 17.5%) involved *Cerebratulus marginatus* Renier, 1804. However, only 16 intraspecific comparisons (8.1%) showed distances above 3.5% and only 2 interspecific comparisons (0.66%) showed distances below 2%.

**Lineus:** The results for the genus *Lineus* were drawn from 1,299 interspecific and 356 intraspecific comparisons and the dataset was represented by nine unique taxonomic lables (i.e., putative species). According to the graph (Fig 3c and 3d), there is a noticeable separation of comparisons between 3–13%, which is the exact range in which barcoding gaps have been reported for other taxa, yet the gap is bordered on both sides by intraspecific and interspecific distances, such that no clear separation of these exists. Interestingly, the vast majority of intraspecific comparisons that result in distances above 5% (starting at 13%) concern *Lineus bilineatus* (Renier, 1804). Indeed, only 14 interspecific comparisons (1.0%) display distance values below 2%, whereas fully 238 intraspecific comparisons (66.8%) show distances above 5%.

**Micrura:** Although the genus *Micrura* has often been referred to as a “mega-genus” in need of taxonomic rearrangements, the 1,004 interspecific comparisons and 768 intraspecific comparisons performed here (for seven unique taxonomic labels) produced a graph that shows an almost perfectly clear and adequately large barcoding gap (Fig 3e and 3f). Apart from the 18 intraspecific comparisons (2.3%) that place in ranges above 17% (all these involve *Micrura fasciolata* Ehrenberg, 1828), all intraspecific comparisons result in distances of 2.5% or below. In addition, all interspecific comparisons resulted in uncorrected *p* distance values of 18% or above, suggesting that, insofar as it is dependent on a barcoding gap, DNA barcoding will allow for accurate identification of specimens within this genus.

### CAOS

The results from the CAOS analyses of selected species groups (see below) is presented in Table 1 and the complete results from the CAOS analyses across all 915 COI sequences are available from the second author upon request. As a first control of the validity of the elevated intraspecific comparisons, the neighbor joining (NJ) tree was interrogated in terms of the distances between clusters of species and lineages within species. If a nominal species showed less than 1% COI distance with other nominal species in the NJ tree, these were suggested to be part of the same species group. In lieu of a more authoritative approach (e.g. [41]) and because clades with multiple taxonomic labels could potentially represent any of the taxa involved (*sensu stricto*), the identity of clades were decided by majority rule of the taxonomic labels—a clade containing 19 specimens of species A and 20 specimens of species B was interpreted as representing species B. Note that, for the vast majority of species, a single distinct clade was present consisting of only one nominal species and with very low internal genetic distances. The complete NJ tree is presented in S1 Fig. Sequences from the following nominal species were assessed because they showed intraspecific variations above 2%: *Cephalothrix filiformis* Johnston, 1828, *C*. *simula*, *C*., *Cerebratulus marginatus*, *Hubrechtella dubia* Bergendal, 1902, *Lineus bilinetaus*, *L*. *ruber* (Müller, 1774), *Micrura fasciolata*, *Nemertopsis flavida* (McIntosh, 1874), *Oerstedia dorsalis*, *O*. *striata*, *Parborlasia corrugatus*, *Ramphogordius sanguineus* (Rathke, 1799), *Riseriellus occultus* Rogers, Junoy, Gibson & Thorpe, 1993, *Tetrastemma melanocephalum* (Johnston, 1837), *T*. *robertianae*, *T*. *roseocephalum* (Yamaoka, 1947), and *T*. *vermiculus* (Quatrefages, 1846).

**Table 1:** Summary statistics for the CAOS analyses of the problematic groups at the species level. Each of the investigated species showed intraspecific variation above 2%, suggesting that DNA barcoding may be hampered by the lack of a distinct barcoding gap. However, each of the different lineages within these species groups possesses diagnostic characters that can aid in the future identification of the lineages (no diagnostic characters where found when comparing the sequences to the entire pool of nemertean taxa; see text for discussion).

Taxon	Number of sequences analyzed	Global, private nucleotides exist *	State: position †	Local, private nucleotides exist for any clade (for each clade) ‡	Clade (state: position) §
*Oerstedia dorsalis*	110	No	N/A	Yes (Yes)	1 (A:66, A:396); 2 (T:243, A:300); 3 (C:57, G:117); 4 (A:54, G:102, A:342, G:504); 5 (G:126, G:291, C:312); 6 (A:36, C:150, G:276, C:474, G:501); 7 (A:12, C:30, A:48, T:117, C:124, T:126, C:193, C:195, T:209, A:261, C:274, T:276, T:282, A:483, G:495); 8 (G:16, A:39, G:87, G:93, G:96, G:147, A:150, C:159, C:186, T:190, A:192, G:213, C:225, A:234, C:243, G:249, G:261, G:288, G:303, A:306, C:309, C:369, C:396, A:456, C:469, T:471, T:475, A:477, T:484, A:486, C:508)
*Cerebratulus marginatus*	7	No	N/A	Yes (Yes)	1 (C:12, G:15, A:27, C:33, A:60, G:72, A:75, C:78, T:111, A:156, A:180, A:192, C:198, A:201, A:210, C:217, A:234, A:237, A:238, G:243, T:261, A:276, A:280, T:303, T:318, C:344, T:363, C:426, A:429, C:435, A:438, A:444, T:477, A:483, T:492, A:507); 2 (A:12, T:21, C:39, A:54, G:60, C:85, T:87, A:96, G:111, T:126, C:133, G:135, C:147, C:162, G:201, C:231, C:243, G:246, G:252, A:261, G:273, T:277, G:306, G:348, A:360, T:396, C:408, A:441, C:466, T:468, A:471, A:495, G:507, G:513); 3 (G:3, T:6, C:22, C:60, C:69, G:87, G:99, A:11, C:114, G:117, T:144, G:147, G:171, C:193, T:195, T:201, C:205, G:207, C:210, G:228, T:234, A:243, T:245, C:249, A:252, T:255, G:258, C:279, C:282, C:294, A:306, G:309, A:315, C:318, C:355, G:363, A:387, A:426, T:435, C:460, T:462, C:474, G:482, T:483, C:508, T:510); 4 (C:15, A:21, A:24, T:27, C:30, G:36, A:39, T:60, G:66, A:72, G:90, T:96, T:99, C:111, T:117, G:126, G:132, G:159, A:168, T:190, G:192, C:198, C:201, A:219, A:220, T:221, A:225, A:228, A:231, T:243, T:249, C:274, T:276, T:280, C:281, A:282, A:286, G:330, G:342, A:345, C:372, A:382, T:384, G:387, A:399, T:400, T:401, T:405, T:406, A:408, G:429, G:448, G:450, G:474, T:475, C:484, T:486, A:492, C:498, C:501)
*Parborlasia corrugatus*	41	No	N/A	Yes (Yes	1 (G:9, A:24, T:27, T:54, C:57, G:60, T:81, C:85, G:111, G:117, T:120, A:123, T:124, G:126, T:150, C:153, A:168, C:174, A:198, T:210, G:231, G:270, T:279, C:294, G:303, A:315, T:328, A:330, C:369, T:381, A:411, T:426, G:510); 2 (A:9, T:15, G:24, C:27, T:39, C:54, A:60, C:81, A:111, A:117, G:123, C:124, T:126. G:150, T:153, T:168, T:174, G:198, C:210, T:231, A:270, C:279, T:294, T:303, G:315, C:328, T:330, T:369, G:381, G:411, G:436, A:510)
*Tetrastemma melanocephalum*	6	No	N/A	Yes (Yes)	1 (A:21, T:22, A:24, A:36, A:48, G:54, A:96, A:126, C:132, G:141, C:150, G:156, T:190, A:192, G:195, C:198, G:207, C:209, G:213, T:214, G:216, G:225, T:228, G:231, T:234, A:240, A:243, G:255, T:261, A:264, G:270, T:274, G:276, G:295, A:300, A:303, G:309, G:315, T:328, A:330, C:333, G:360, T:405, A:429, T:441, T:453, A:456, C:460, T:469, A:471, C:475, T:480, T:501, A:510); 2 (G:6, A:12, A:87, C:90, T:108, G:132, G:144, G:168, A:183, G:208, G:237, G:243, G:273, T:282, C:291, A:363, G:381, G:396, G:405, A:420, G:447, G:462, C:466, T:468, A:477); 3 (G:15, A:18, C:37, T:39, C:45, T:72, A:108, A:111, G:123, T:132, A:144, G:159, T:177, A:189, G:201, C:210, C:217, T:243, A:258, C:270, C:294, A:399, A:405, G:408, G:423, A:438, A:457, A:504, T:513)
*Tetrastemma robertianae*	6	No	N/A	Yes (Yes)	1 (C:1, T:3, T:6, G:18, T:48, A:60, T:72, G:87, G:90, T:99, A:102, G:111, G:117, G:132, A:141, C:159, T:168, A:171, G:189, C:193, T:195, T:201, T:210, C:217, T:219, A:234, A:237, A:240, T:243, T:255, A:258, A:261, T:264, T:270, A:273, T:279, A:282, A:291, A:300, A:309, A:318, T:327, G:342, A:345, C:348, T:363, G:385, A:396, A:399, C:405, G:408, G:420, G:423, G:435, A:438, A:444, A:447, A:453, A:462, C:463, A:468, C:469, T:471, G:483, G:492, A:513); 2 (T:1, G:3, A:6, T:18, A:48, T:60, A:72, A:87, T:90, A:99, G:102, T:111, A:117, T:132, G:141, T:159, G:168, G:171, A:189, T:193, G:201, A:210, T:217, A:219, G:234, G:237, T:240, G:243, G:255, G:258, T:261, A:264, C:270, T:273, A:279, T:282, T:291, G:300, T:309, T:318, A:327, T:342, T:345, A:348, A:363, A:385, G:396, G:399, A:408, A:423, T:435, G:438, T:444, G:447, T:453, G:462, T:463, G:468, T:469, A:471, A:483, T:492, G:513)

* Result based on comparison of the entire pool of sequences for the taxon listed versus all sequences from the 915-taxon dataset.

^†^ Relative position of the nucleotides based on the alignment used in the present study.

^‡^ Result based on comparison of each clade found in the NJ analysis versus the remainder of sequences with identical taxonomic labels.

^§^ Clade number refers to the numbers in the NJ tree (S1 Fig).

After detailed examination of the NJ tree, several misidentifications could be determined; some representatives of taxa that showed high intraspecific distances were recovered in clades of other species and with very low genetic distances, often zero percent. For example, a single specimen of *Hubrechtella dubia* placed within a clade of numerous specimens of *Cephalothrix rufifrons* (Johnston, 1837) and with zero percent distance, strongly suggesting that this specimen was misidentified or that the sequence is somehow contaminated (this was also corroborated by a BLASTn analyses in each case); the remaining specimens of *H*. *dubia* form a separate clade with low genetic distances (average 0.51% ± 0.32 uncorrected-*p* distance) and these were therefore not included in the CAOS analyses. By contrast, *Cerebratulus marginatus* places in four different clades in the tree and half of these also contain other taxonomic labels. Because none of these clades hold a majority of specimens for *C*. *marginatus*, all of the separate lineages were analyzed with CAOS. As a result of the initial NJ tree examinations, only *Cerebratulus marginatus*, *Oerstedia dorsalis*, *Parborlasia corrugatus*, *Tetrastemma melanocephalum* and *Tetrastemma robertianae* seem to present some form of evidence pointing to the presence of more than one distinct lineage in the NJ tree. None of the problematic species groups presented global diagnostic characters when compared to all 915 COI sequences of the full data set. By contrast, within each of the five smaller datasets for the nominal species, each lineage showed diagnostic characters that would allow for their separation from other lineages. For example, the eight different lineages labeled as *Oerstedia dorsalis* present in the NJ tree each shows between one and 31 diagnostic characters that allow for identification of the specific clade within a pool of *O*. *dorsalis* sequences (Table 1). The lineages for the remaining species showed between and 25 and 66 diagnostic characters, which suggests that there is support for the separation of these lineages into species-level taxa.

### Species delimitation

Unsurprisingly, given the disparity of branch lengths across the input BEAST tree, the GMYC multiple threshold model had a slightly better fit to the data than the single threshold model, the former resulting in an overall maximum likelihood (ML) score of 7742.761. The GMYC analysis suggested that 115–118 ML clusters (i.e., species groups with necessarily more than one representative sequence) were present in the dataset, but that fully 399–402 ML entities (i.e., delimited species, inclusive of “species” for which only a single sequence was represented) were represented among the data (likelihood ratio test p-value = 0). On the one hand, the Bayesian estimation of the PTP analysis suggested that between 227–371 species were present in the dataset (mean species number: 307.52; acceptance rate: 0.439886; merge value: 249608; split value 250392). On the other hand, the ML solution recovered by the PTP analysis suggested that 185 species exist in the dataset. The TCS haplotype analyses predicted that 190 OTUs were present at a connection limit of 95% and that 214 OTUs were present at 98%.

**Fig 4:**
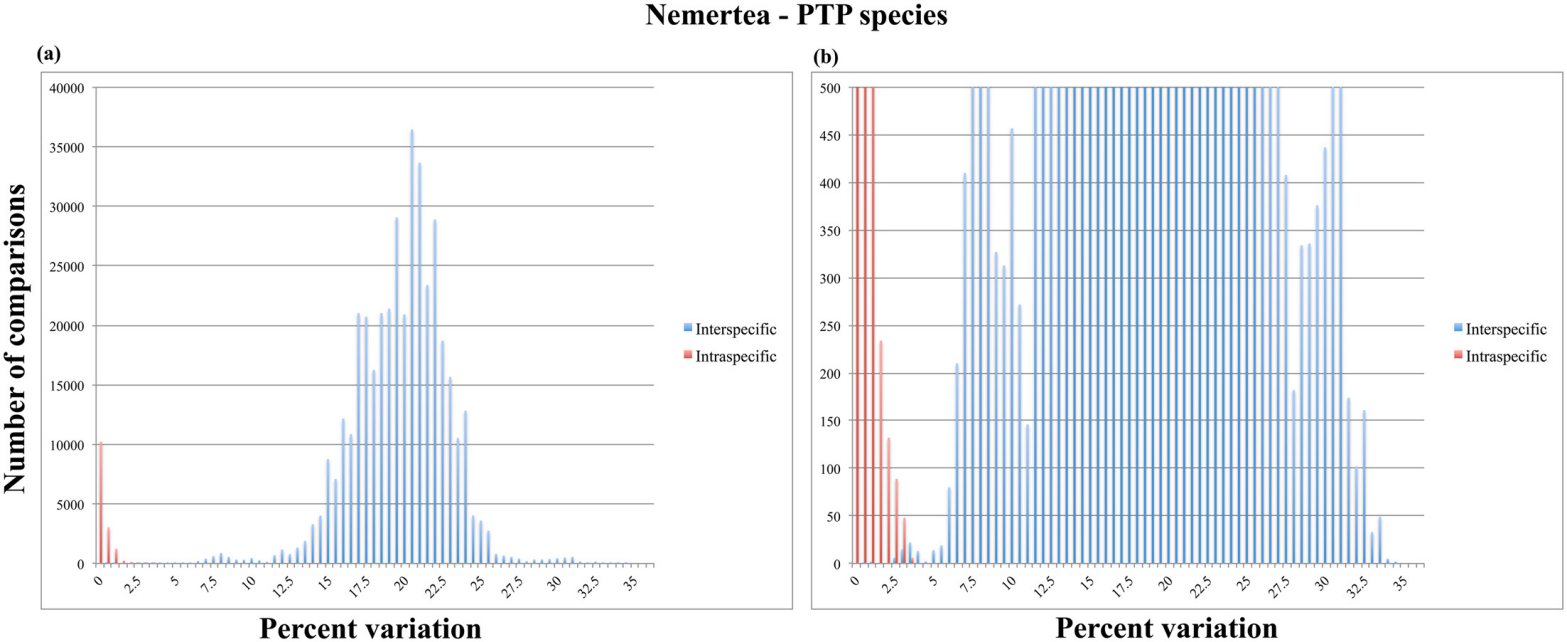
Result from the manual calculations of intraspecific (red) and interspecific (blue) COI distances using species affiliation determined by the ML solution in PTP. Note the presence of a short barcoding gap at ~3–5%, which is further discussed in the text. A, Nemertea, full view of the chart with the x-axis set above the upper limit of the number of comparisons within the dataset; B, Nemertea, enlarged view of the barcoding gap region with the x-axis set to a maximum of 500 comparisons.

Given the disparate numbers of predicted species when compared to the number of taxonomic labels, we also assessed whether or not a barcoding gap presents itself using more objectively determined species affiliations. To this end, the full nemertean dataset was re-analyzed using species affiliations as determined by the ML solution in PTP; this scheme revealed 185 species, which approaches the number of taxonomic labels present in the dataset. The results (Fig 4a and 4b) suggest that a barcoding gap does exist, albeit with a rather narrow range, when species are objectively assigned.

## Discussion

Barcoding gaps, or at least tendencies towards such gaps, are present in most of our datasets and are generally expressed, with varying width, between 4–10% COI variation—for example, for the full Nemertea dataset, there is a clear decrease in the number of comparisons that show between 4–5% variation but the number of comparisons quickly increase on either side of this “gap” (see Fig 1). This suggests that DNA barcoding works for most nemertean taxa, insofar as a barcoding gap is present and assuming that the taxonomic labels are correct. The bulk part of nemertean taxonomy is built on elderly descriptions that often do not always lead to identifications of the same certainty as the requirements of today [2]. With vague and nonspecific descriptions like "a small brown worm with a dorsal white median line” (*Oerstedia dorsalis* [Abildgaard 1804]), it is clear that many subsequent biologists have been tempted to use that name whenever they found a specimen resembling the vague description. In this way, some species names end up as "taxonomic trash cans" where the name does not correspond to one single species, however defined. Consequently, there are many cases where barcode sequences are tagged with the same name while, in fact, representing different species (see e.g. [42,43]). This is particularly true for some of the nominal species in this study (e.g. those of *Oerstedia*, *Lineus*, *Cerebratulus*), which is why some of the intraspecific variations shown in our results are in fact interspecific divergences. This is strongly supported by all of our species delimitation analyses (GMYC, PTP and TCS), which all indicate that a higher number of species are represented within our dataset—indeed, the number of predicted species ranged between 185 (for the ML solution using PTP) and 400 (using GMYC under the multiple threshold model). Importantly, an upper limit of 3% intraspecific COI variation is often encountered for the limited set of nemertean specimens for which clear-cut morphological characters exist (authors’ personal observation). In other words, a COI variation above 3% is likely to suggest that the compared sequences are derived from different species—this has also been suggested for other taxa (e.g. [44]). When using objectively assigned species affiliations based on a Poisson Tree Process analysis, our results indicate that a short barcoding gap exists between ~3–5% (although a few outlier values for interspecific comparisons nest among the intraspecific comparisons). It seems likely that, assuming accurate specimen identification, intraspecific genetic variation within Nemertea can be defined by a 3%-rule, as suggested for other groups.

Although we believe that DNA barcoding is a useful and applicable approach to identifying nemertean specimens, we submit that there will be errors as long as the taxonomy is not fully resolved. However, the same problem would appear using external morphology to identify individuals particularly when it comes to cryptic species. In the latter case, we would not even suspect or receive any red flags regarding identification problems, which is the case when using DNA. Minimally, the results presented here will aid in future taxonomic revisions of the species involved and may offer guidance as to which lineage within a nominal species represents the taxon *sensu stricto*. DNA barcoding using only the COI locus holds potential for identification of nemertean specimens within certain clades but overall values suggest that identifications based on COI may be prone to error due to the occupation of the barcoding gap by both interspecific and intraspecific distance values. One of the most revealing trends evinced here is that interspecific divergence values for the entire pool of sequences are typically better behaved than intraspecific distance values. Although such an assertion assumes equal taxon representation within the dataset, an assumption that is clearly violated in most cases, the proportion of uncommonly high intraspecific variations is much greater than the proportion of uncommonly low interspecific variation within the data shown here. What does this mean in reality? The answer to this question is dual depending on whether or not the taxonomic labels are assumed to be correct: if the labels are trusted then these results may indicate that insipient speciation is abundant within the phylum (due to e.g. geographic or reproductive isolation; [45,46]) or that high levels of cryptic speciation followed by incomplete lineage sorting has shaped genetic compositions [18]. This would, in effect, render DNA barcoding an inadequate approach for identification of these taxa but could still be a valuable tool for lower taxonomic ranks that show a decrease in the amount of high intraspecific variation. By contrast, if the taxonomic labels associated with several of the sequences in the commonly used barcode repositories are not trusted but, instead, further scrutinized in a cogent manner, then the data indicate that DNA barcoding is a valuable tool for identification of nemerteans. A closer survey of the sequences for some of the problematic taxa shows that the majority of these are identical to sequences associated with disparate taxonomic labels, suggesting a misidentification and/or mislabeling. When these sequences are removed, the barcoding gap becomes increasingly distinct and sufficiently sized (data not shown). Many of the problematic sequences are found in certain taxonomic groups (see Results). It is therefore important to have even a modest *a priori* knowledge concerning the taxonomy of the specimens when identifying unknown samples with a barcoding approach. The search can then be restricted to a lower taxonomic level. Still, it remains that a smaller proportion of interspecific distances fall within the typical range of intraspecific variation, a confounding factor that deserves future attention.

Another obstacle facing DNA barcoding is the mislabeling of sequences in the main DNA sequence repositories—a problem also pointed out by Ekrem et al [47]. Much like assuming correct taxonomic labels, sequence data are seldom scrutinized in a manner that allows for the discrimination of contaminations; this is becoming increasingly true with the development of large datasets generated by high-throughput sequencing efforts as inspection of individual gene sequences becomes more computationally challenging.

## Supporting Information

S1 Fig Midpoint-rooted neighbor joining tree derived from the full 915-sequence dataset. The tree was used to guide the separation of lineages for the CAOS analyses of smaller datasets (see text for further details).
(PDF)

S1 Table List of specimens for which COI sequences were newly generated and used in the present study. (XLSX)
