# Supplementary figures and images for: Evaluating the Utility of Single-Locus DNA Barcoding for the Identification of Ribbon Worms (Phylum Nemertea)
Source: PLoS One. 2016 May 12;11(5):e0155541. doi: 10.1371/journal.pone.0155541 (PMC4865114; doi:10.1371/journal.pone.0155541)

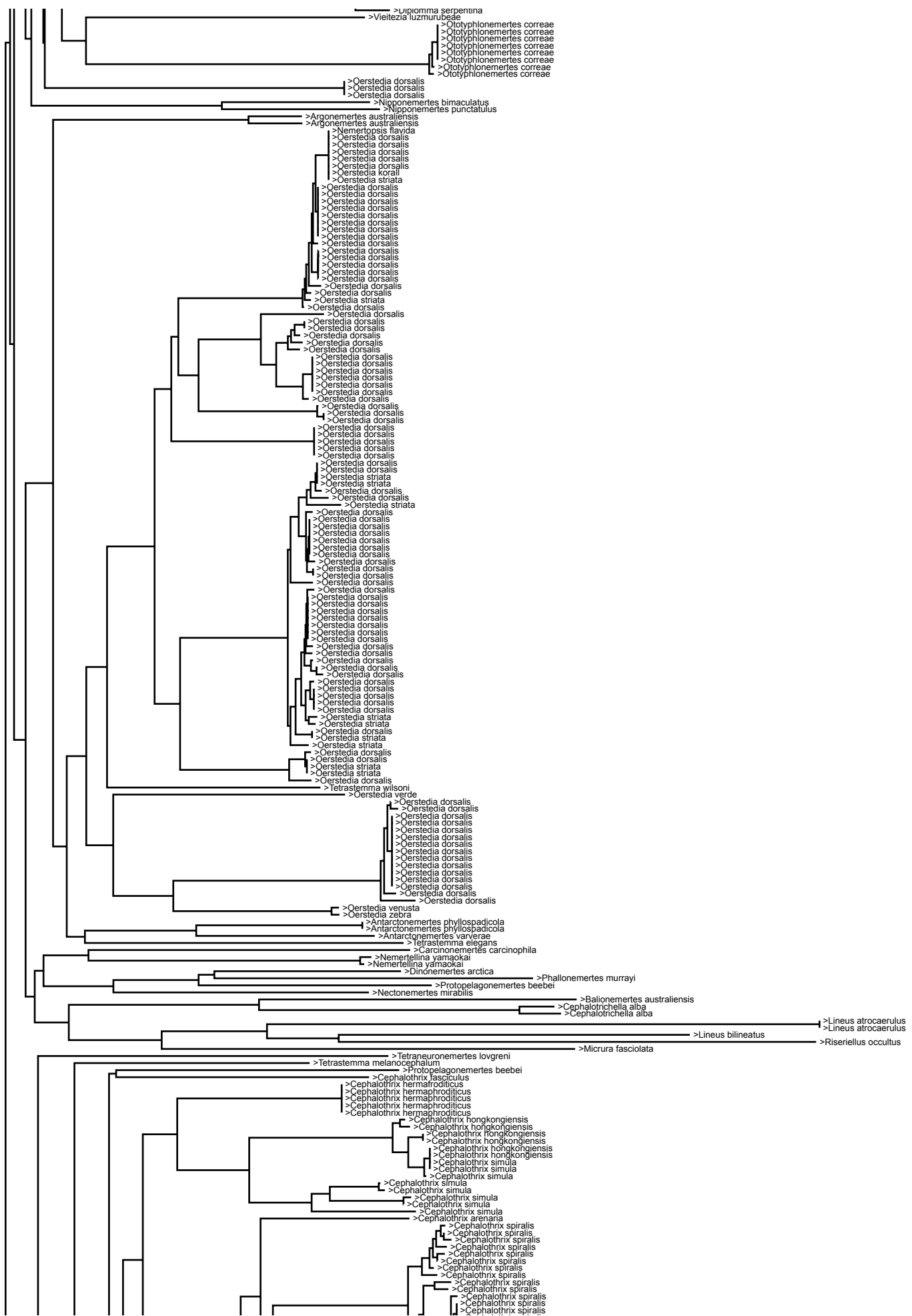

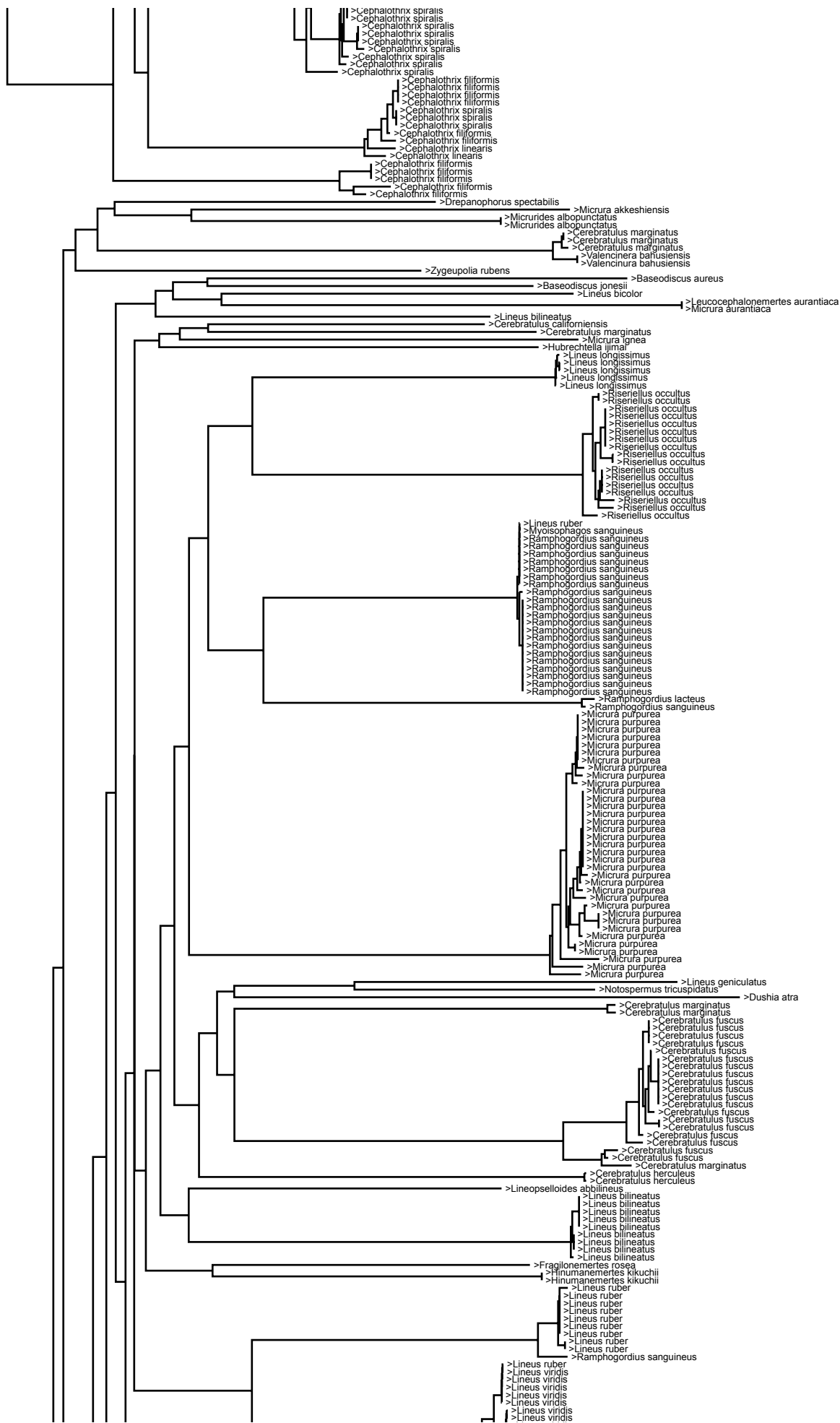

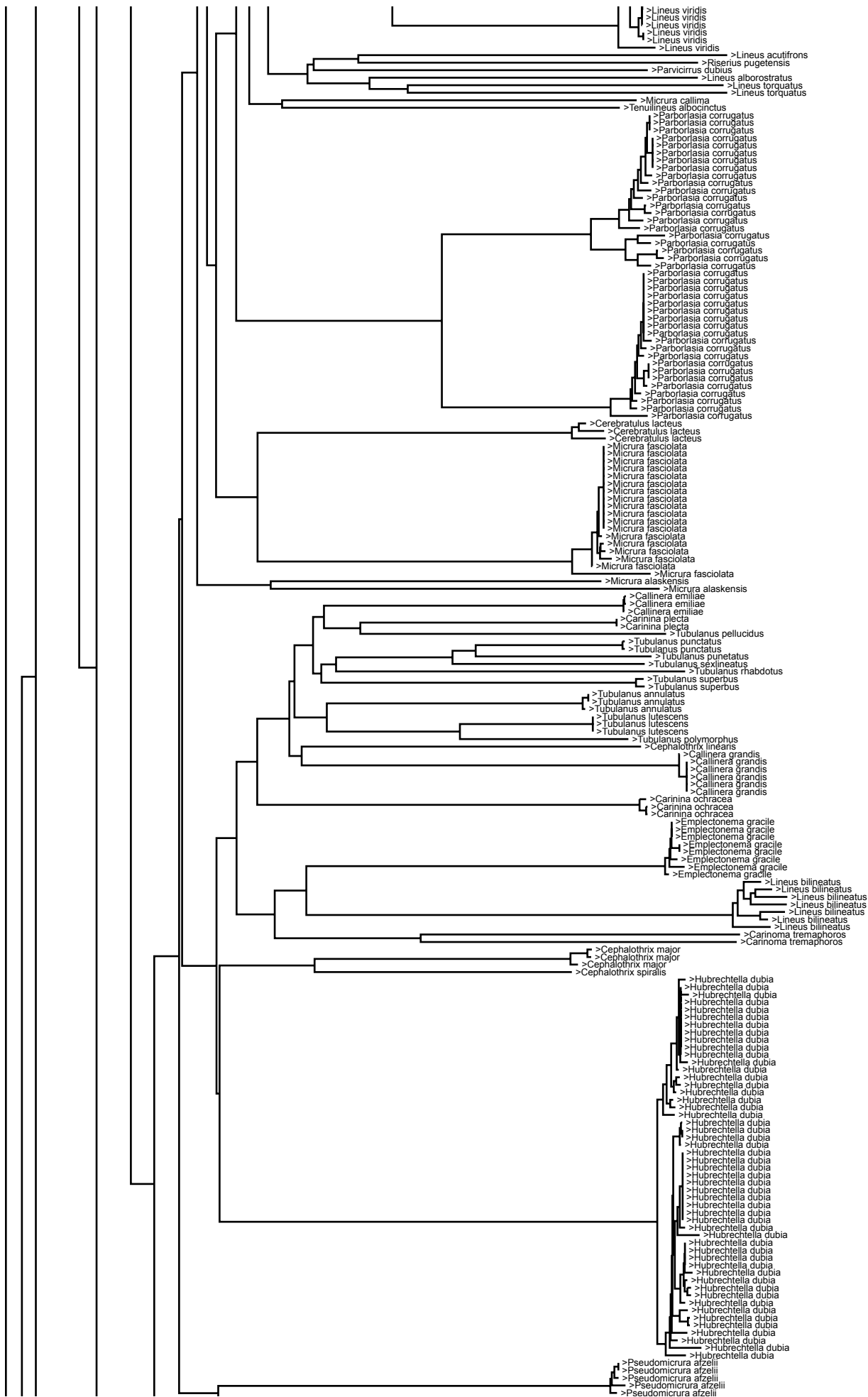

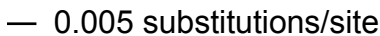

Supplement: S1 Fig — The tree was used to guide the separation of lineages for the CAOS analyses of smaller datasets (see text for further details). (PDF) [file pone.0155541.s001.pdf]
